# Cooking-Related Skills and Food Sustainability-Related Practices: A Systematic Review

**DOI:** 10.3390/foods15111899

**Published:** 2026-05-28

**Authors:** Daniele Nucci, Flavia Pennisi, Antonio Pinto, Vincenza Gianfredi, Carlo Signorelli

**Affiliations:** 1 National PhD Programme in One Health Approaches to Infectious Diseases and Life Science Research, Department of Public Health, Experimental and Forensic Medicine, University of Pavia, 27100 Pavia, Italy; pennisi.flavia@hsr.it; 2 Struttura Semplice Dipartimentale Igiene Alimenti e Nutrizione, Dipartimento di Igiene e Prevenzione Sanitaria, Agenzia di Tutela della Salute (ATS) Brescia, Via Duca degli Abruzzi, 15, 25124 Brescia, Italy; 3 Faculty of Medicine, Vita-Salute San Raffaele University, 20132 Milan, Italy; pinto.antonio@hsr.it (A.P.); signorelli.carlo@hsr.it (C.S.); 4 Department of Cardiac Thoracic Vascular Sciences and Public Health, University of Padua, 35131 Padova, Italy

**Keywords:** cooking skills, healthy diet, mediterranean diet, food sustainability, food behaviors, home cooking, culinary education, teaching kitchen

## Abstract

Cooking-related skills, encompassing practical food preparation skills and behaviors associated with food management, have been increasingly recognized as potential determinants of healthier and more sustainable dietary behaviors. However, the extent to which cooking skills and related practices contribute to food sustainability-related practices remains unclear. This systematic review aimed to assess the relationship between cooking-related skills and food sustainability-related practices. The literature search was conducted in PubMed/MEDLINE, Scopus and Embase and updated on 25 February 2026. Eligible studies included peer-reviewed studies examining associations between cooking-related skills (including cooking skills, home cooking, culinary education, and teaching kitchen interventions) and food sustainability-related practices (such as diet-related greenhouse gas emissions). Eligible studies included quantitative and qualitative designs conducted among adult populations. Risk of bias was assessed using design-specific appraisal tools. Results were reported according to PRISMA 2020 guidelines. Overall, 28 studies published between 2015 and 2026 (comprising 32,211 participants) were included. Of these, 16 studies were cross-sectional, 9 used pre–post intervention designs, and the remaining evidence included qualitative, mixed-methods, and randomized or cluster-randomized designs. Food waste outcomes were assessed in 19 studies, of which 15 reported favorable associations between cooking-related skills, home cooking, or cooking-based interventions and lower food waste or better food waste prevention behaviors. Diet quality outcomes were assessed in 12 studies and generally suggested favorable associations with Mediterranean diet adherence, fresh food consumption, and lower processed or ultra-processed food intake. Cooking-related skills may represent a promising behavioral pathway to promote healthier and more sustainable food practices. Nevertheless, stronger evidence from well-designed longitudinal and experimental studies is needed to clarify causal relationships and better inform public health strategies aimed at supporting sustainable food systems.

## 1. Introduction

Cooking-related or culinary skills broadly encompass practical food preparation skills, technical abilities, knowledge, and behaviors associated with food management, including the ability to select, combine, prepare, cook, and present foods, particularly when based on fresh and minimally processed ingredients [1,2]. Food literacy represents a broader construct than cooking skills. It includes the knowledge, skills, and critical abilities needed to plan, select, prepare, and consume foods in ways that support individual health, social well-being, and, increasingly, environmental sustainability. From this perspective, cooking skills may be considered a practical and behavioural component of food literacy, but they do not fully capture the broader cognitive, planning, and decision-making dimensions involved in food choices [3,4]. The ability to prepare and cook meals from scratch has been associated with greater dietary variety, higher consumption of fruits, vegetables, and legumes, lower intake of ultra-processed foods, and improved overall diet quality [5,6]. Nevertheless, home cooking-related skills are undergoing a progressive decline across high- and middle-income countries, a trend interpreted as an expression of the culinary transition [7].

This transition reflects a shift away from traditional home-food preparation practices toward increased reliance on convenience foods, ultra-processed products, ready-to-eat meals, and out-of-home meal consumption [8]. In parallel with the nutrition transition, characterized by a shift toward energy-dense, highly processed diets [9], the culinary transition captures the progressive loss of practical cooking knowledge and skills across generations [10,11]. Cooking skills, particularly those related to home food preparation, have emerged as an important area of interest in public health nutrition [12]. This growing interest occurs within a conceptual shift in global health toward more integrated and systemic approaches. This perspective aligns closely with the One Health and Planetary Health frameworks, which emphasize the interconnectedness of human and environmental well-being. The One Health approach recognizes the interdependence of human, animal, and environmental health, advocating for integrated strategies to address complex health challenges [13]. Complementarily, the Planetary Health perspective highlights that human health is strictly dependent on the state of natural systems, underscoring the urgent need to transition toward more sustainable food systems [14]. The transition toward healthier and more sustainable food systems represents one of the foremost public health challenges. The global agri-food system accounts for approximately one-third of total greenhouse gas (GHG) emissions and is a leading driver of land-use change, freshwater depletion, and biodiversity loss [15].

Moreover, food systems are increasingly recognized as central to achieving the United Nations Sustainable Development Goals (SDGs). As highlighted by Johan Rockström and Pavan Sukhdev, food represents an interconnected domain that directly or indirectly influences all SDGs, including those related to health, environmental sustainability, social equity, and responsible consumption [16]. On the other hand, suboptimal dietary patterns are a major contributor to the global burden of non-communicable diseases [17]. In this framework, sustainable diets—defined by the Food and Agriculture Organization (FAO) as “diets with low environmental impacts that contribute to food and nutrition security and to healthy life for present and future generations”—have gained increasing attention [18,19]. Among these, the Mediterranean diet has been widely recognized as a dietary pattern that combines nutritional adequacy with low environmental impact [20]. Sustainability of the Mediterranean diet is widely attributable to its predominantly plant-based composition, high reliance on seasonal and locally sourced foods, limited intake of animal products, and emphasis on biodiversity [21], all of which contribute to lower environmental impacts [22]—such as reduced greenhouse gas emissions, land use, and water consumption—while simultaneously promoting human health. In addition, it encourages practices that help reduce food waste, for example, through the use of simple ingredients, culinary creativity, and the reuse of leftovers [23]. Furthermore, the Mediterranean diet embodies a strong cultural heritage, encompassing not only traditional food practices but also food-related skills and the intergenerational transmission of culinary knowledge [24].

However, the promotion of healthy and sustainable dietary patterns is often constrained by a range of socio-economic, cultural, and environmental factors [25,26,27]. Particularly, individual-level determinants of food behavior, such as cooking skills, have gained increasing attention as potentially modifiable factors [28,29]. In this review, sustainable food behaviors refer to individual or household food-related practices that may contribute to healthier and more sustainable food systems. These include, but are not limited to, reducing household food waste, using fresh, seasonal, local, or minimally processed foods, limiting processed and ultra-processed food consumption, and adhering to sustainable dietary patterns such as the Mediterranean diet. Despite growing interest in this field, the extent to which cooking-related skills contribute to food sustainability-related practices and food behaviors remains unclear, and the existing evidence appears fragmented. Therefore, the aim of the present systematic review was to synthesize the available evidence on the association between cooking-related skills and sustainable food behaviors in adult populations.

## 2. Methodology

### 2.1. Study Design and Reporting Standards

This systematic review was conducted following the guidelines of the Cochrane Collaboration [30], and the results were reported in accordance with the Preferred Reporting Items for Systematic Reviews and Meta-Analyses (PRISMA) 2020 guidelines [31]. The aim of the review was to synthesize the available evidence on the association between cooking-related skills and food sustainability-related practices within a public health and planetary health perspective. The research protocol was defined in advance and shared among the research team. Therefore, the protocol was registered in the international database of prospectively registered systematic reviews in Zenodo (registration date: 19 November 2025, https://doi.org/10.5281/zenodo.17649618).

### 2.2. Search Strategy

A comprehensive literature search was simultaneously conducted in three electronic databases: PubMed/MEDLINE, Scopus, and EMBASE, on 19 November 2025 and updated on 25 February 2026. The search strategy combined Medical Subject Headings (MeSH) and free-text keywords related to cooking skills (exposure) and food sustainability-related practices (outcome). Keywords included terms such as cooking skills, culinary skills, home cooking, culinary medicine, teaching kitchen, and terms related to food sustainability-related practices, including food waste, sustainable diet, Mediterranean diet, organic food, seasonal food, and processed food consumption.

The search strategy was designed to maximize sensitivity by incorporating synonyms and alternative terminology for the key exposure and outcome domains. In addition to database searches, supplementary strategies were applied, including manual screening of reference lists of relevant articles, examination of studies listed as “related articles” in PubMed, and targeted searches of publications from key authors identified during the screening process. Grey literature sources, including reports from public health agencies, government nutrition programs, international organizations, and non-peer-reviewed institutional documents, were not systematically searched. This decision was consistent with the predefined scope of the review, which aimed to synthesize peer-reviewed empirical studies providing sufficient methodological information for standardized data extraction and risk-of-bias assessment. The full search strategy, for each database, is reported in Supplementary Table S1. The searches were performed blindly by two researchers (VG and DN), and an equal number of records were retrieved.

### 2.3. Eligibility Criteria

Studies were deemed eligible for inclusion if they fulfilled a predefined set of criteria. The eligibility criteria for study inclusion were defined according to the population, exposure, outcomes, and study design of interest. Specifically, studies were considered eligible if they included adult participants aged 18 years or older and examined cooking-related skills or activities, such as cooking skills, culinary skills, home cooking practices, culinary medicine programs, teaching kitchen interventions, or other cooking-based educational initiatives. Eligible studies were required to assess food sustainability-related practices as outcomes, including indicators such as food waste reduction, sustainable food choices (e.g., consumption of seasonal, organic, or minimally processed foods), and adherence to sustainable dietary patterns, particularly the Mediterranean diet. Both quantitative and qualitative research designs were considered eligible, provided that the studies were published as peer-reviewed scientific articles. Studies were excluded if they involved participants younger than 18 years, were not published as peer-reviewed scientific articles, or were not available in English, unless a reliable translation of the full text was available. Accordingly, grey literature sources, such as technical reports, policy documents, government program evaluations, conference proceedings, and non-peer-reviewed institutional documents, were not eligible for inclusion. A detailed description of inclusion/exclusion criteria is reported in Supplementary Table S2.

### 2.4. Study Selection

All records retrieved from the electronic databases were imported into a reference management system (EndNote X9, Clarivate, Philadephia, PA, USA), and duplicates were removed automatically prior to screening. Titles and abstracts were screened using the Rayyan tool (Rayyan System Inc., Cambridge, MA, USA) independently by two reviewers to identify potentially eligible studies. Full texts of all potentially relevant articles were then assessed for eligibility according to the predefined inclusion and exclusion criteria. Disagreements between reviewers were resolved through discussion and consensus. If consensus could not be reached, a third senior reviewer (V.G.) was designated to act as an arbiter and make the final decision.

### 2.5. Data Extraction

Data extraction was conducted independently by two reviewers using a predefined and pilot-tested data extraction form developed in Excel (Microsoft Excel^®^ for Microsoft 365, Redmond, WA, USA, 2019). To improve the quality of data extraction, the spreadsheet was pre-tested on 3 randomly selected studies. The following information was extracted from each included study: bibliographic details (author, year of publication, and country), study design and methodological characteristics, population characteristics (including sample size, age, and gender distribution), definition and measurement of the exposure related to cooking skills or related constructs, outcome measures related to food sustainability-related practices, and the statistical method. Main study results were summarized, reporting effect estimates such as odds ratios (ORs) and 95% confidence intervals (CIs) when available, or narratively describing findings when quantitative data were not reported. When applicable, we extracted the list of covariates included in the statistical adjustment of models (e.g., sociodemographic or behavioral variables). Additionally, any health-related outcomes—if reported as secondary or exploratory findings—were noted. Finally, information on potential conflicts of interest and sources of funding was systematically recorded for each study. Discrepancies in extracted data were resolved through discussion between the reviewers until consensus was reached. If consensus could not be achieved, a third senior reviewer (V.G.) was available to adjudicate.

### 2.6. Outcomes

In this review, food sustainability was not considered as a single homogeneous outcome, but as a multidimensional construct including environmental/resource-use, health/nutritional, and economic or food management-related dimensions. Accordingly, we considered selected food sustainability-related practices and outcomes. Environmental and resource-use outcomes included household food waste prevention, measured food waste, leftover use, and, when available, diet-related environmental impact indicators such as greenhouse gas emissions. Health and nutritional outcomes included diet quality, adherence to sustainable dietary patterns such as the Mediterranean diet, consumption of fresh, seasonal, organic, or minimally processed foods, and reduced consumption of processed or ultra-processed foods. Economic or food management-related dimensions included practices such as meal planning, efficient use of ingredients, food storage, and household food resource management, when these were assessed in the included studies. Both self-reported and objectively measured outcomes were considered eligible for inclusion.

### 2.7. Quality Assessment

The methodological quality of the included studies was evaluated using appropriate appraisal tools depending on the study design.

Observational studies were assessed using the Newcastle–Ottawa Scale (NOS) or an adapted version (NOS-xs) suitable for cross-sectional study [32]. The NOS-xs tool evaluates potential sources of bias across three main domains: selection of the study sample, assessment of exposure and outcomes, and control of confounding factors. The NOS-xs assigns a maximum of nine stars, with higher scores indicating lower risk of bias. According to the proposed classification, studies scoring 7–9 stars were considered at low risk of bias, 4–6 stars at moderate risk, and 0–3 stars at high risk of bias.

The methodological quality of quasi-experimental studies was assessed using the Joanna Briggs Institute (JBI) Critical Appraisal Checklist for Quasi-Experimental Studies [33]. This tool evaluates the risk of bias across nine domains, including the clarity of the temporal relationship between intervention and outcome, similarity of participants in comparisons, similarity of care apart from the intervention, presence of a control group, repeated outcome measurement before and after the intervention, completeness of follow-up, consistency of outcome measurement, reliability of outcome assessment, and appropriateness of the statistical analysis. Each item was rated as Yes, No, Unclear, or Not Applicable, as appropriate for the design of each included study. For single-group pre-post studies, items specifically referring to between-group comparability were considered not applicable. To improve consistency, review-specific decision rules were defined a priori for the interpretation of each domain. An overall methodological quality rating was then assigned based on the proportion of applicable checklist items rated as “Yes”, with studies classified as high, moderate, or low methodological quality. Quality assessment was conducted independently by two reviewers, and discrepancies were resolved through consensus.

The methodological quality of qualitative studies was assessed using the Joanna Briggs Institute (JBI) Critical Appraisal Checklist for Qualitative Research [34]. This tool evaluates the methodological rigor and credibility of qualitative studies across ten domains, including the congruity between research methodology and research question, adequacy of data collection and analysis, consideration of researcher reflexivity, ethical approval, and representation of participants’ voices. Each item was rated as “yes”, “no”, “unclear”, or “not applicable”.

For randomized trials, the Cochrane Risk of Bias tool (RoB 2.0) [35] was employed, which assesses potential sources of bias across domains such as the randomization process, deviations from intended interventions, missing outcome data, measurement of the outcome, and selection of the reported result.

### 2.8. Data Synthesis

A narrative synthesis was undertaken due to expected heterogeneity in study designs, populations, and outcome measures. For synthesis purposes, outcomes were grouped thematically into food waste and household food resource management, sustainable diets and diet quality indicators, and direct environmental sustainability indicators. The high heterogeneity in outcome measurement and data reporting prevents us from performing a meta-analysis.

## 3. Results

### 3.1. Selection Process

**Figure 1:**
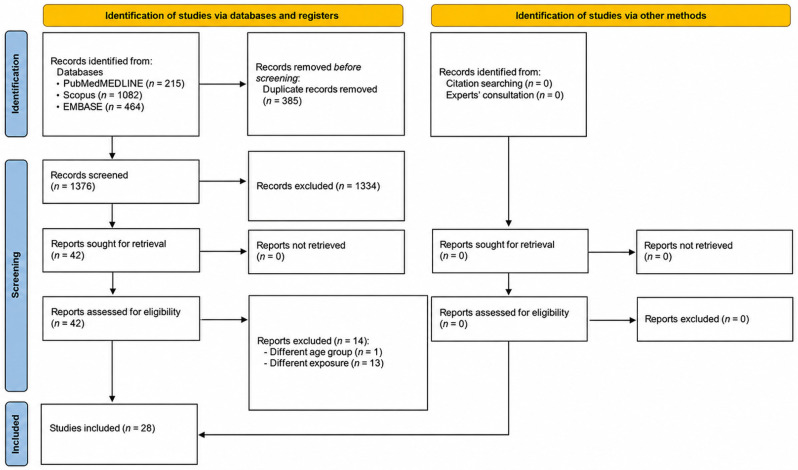
PRISMA 2020 flow diagram showing the selection process.

The literature search identified a total of 1761 records across the three electronic databases searched: PubMed/MEDLINE (n = 215), Scopus (n = 1082), and EMBASE (n = 464). After removal of 385 duplicate records, 1376 unique records remained and were screened based on title and abstract. Of these, 1334 records were excluded because they did not meet the eligibility criteria. A total of 42 articles were therefore retrieved for full-text assessment. All reports were successfully obtained and evaluated for eligibility. Following full-text review, 14 studies were excluded, including one study conducted in a different age group [36] and 13 studies that did not evaluate the exposure of interest [37,38,39,40,41,42,43,44,45,46,47,48,49]. No additional studies were identified through other sources, such as citation searching or expert consultation. Consequently, 28 studies met the inclusion criteria and were included in the qualitative synthesis. The selection process is depicted in Figure 1.

### 3.2. Main Characteristics of Included Studies

**Figure 2:**
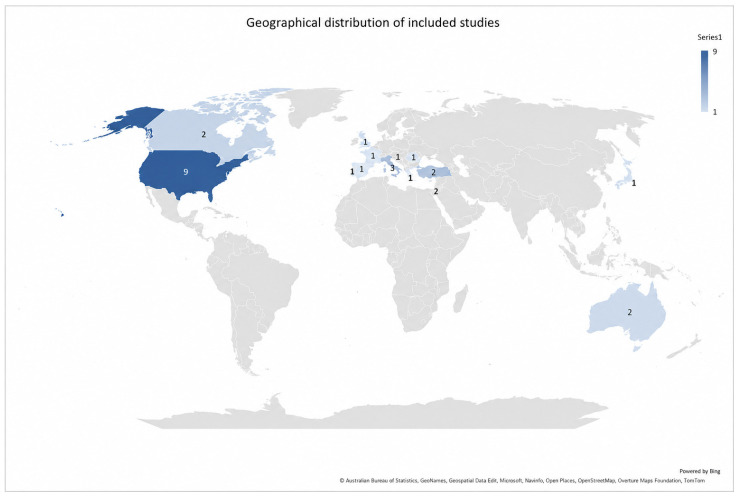
Geographical distribution of the included studies based on the country where the studies took place. For multicountry studies, each country involved in the study was represented on the map. Therefore, Finkelstein et al. [51], which was conducted in both the United States and Israel, is displayed in both countries for descriptive mapping purposes only. The study was counted once in the total number of included studies and in the qualitative synthesis.

A total of 28 studies were included in the review, published between 2015 [50] and 2026 [51,52]. Overall, the evidence base was geographically diverse (Figure 2), with studies conducted in the United States (n = 8), Turkey (n = 3), Italy (n = 3), Australia (n = 2), Canada (n = 2), and single studies from Greece, France, Spain, Portugal, Austria, Romania, Japan, Israel, and Scotland; one study was conducted across both the United States and Israel [53]. For the geographic map, multicountry studies were represented in each country where the study was conducted. Accordingly, Finkelstein et al. [53] was displayed in both the United States and Israel. This representation was used only to describe the geographic settings of the included evidence and did not affect the number of unique studies included in the review or the qualitative synthesis. Study designs were predominantly observational, with 16 cross-sectional studies [51,54,55,56,57,58,59,60,61,62,63,64,65,66,67,68], while 9 studies used a pre-post intervention design. The remaining evidence comprised one cluster-randomized pilot trial [69], and two qualitative studies [53,70], one qualitative study nested within a randomized trial [53], and one mixed-methods multi-study investigation [67]. Intervention-based studies represented an important subset of the included evidence. These studies defined the exposure as participation in structured cooking education, teaching kitchen, culinary medicine, or coaching programs, including online nutrition and cooking courses, workshop-based cooking classes, experiential teaching kitchen programs, individualized culinary coaching, and community- or university-based cooking interventions. In these studies, the exposure was therefore represented by participation in an educational or behavior-change program rather than by a single baseline cooking skill score. Overall, intervention-based studies most commonly reported changes in diet quality, fresh food consumption, processed food intake, food waste awareness, or food waste prevention behaviours. However, most interventions used pre–post designs, often without control groups, and frequently relied on self-reported outcomes; therefore, the findings should be interpreted as preliminary evidence of potential benefit rather than definitive evidence of effectiveness. No school-based programs involving children or adolescents were included, as the eligibility criteria were restricted to adult populations. Educational interventions included in this review, therefore, mainly refer to adult, community, university, teaching kitchen, or culinary medicine settings.

The total number of participants analyzed across all included studies was 32,211, although sample size varied markedly, ranging from 9 [70] to 11,982 participants [60].

The populations investigated were heterogeneous but largely adult-based, in line with the review question. Several studies included general adult populations, household food shoppers or meal providers, and adults responsible for food purchasing and cooking. Other studies focused on more specific subgroups, including university students, university staff, low-income families, parents of young children, community-based participants, adults with overweight or obesity, family physicians, and adult women from a population cohort. Where sex distribution was available, women were generally overrepresented, with a median female proportion of approximately 74%. Several studies included predominantly female samples, and some were conducted entirely or almost entirely among women or female household food preparers. However, most studies did not report sex-stratified results, preventing any formal assessment of sex as a potential effect modifier. Participant age also varied substantially, from university-age young adults to middle-aged and older community samples, indicating that the literature spans multiple stages of adult life. Details are reported in Table 1.

### 3.3. Conceptual Classification of Exposures: Cooking Skills, Food Literacy-Related Constructs, and Food Behaviors

Exposure definitions varied substantially across the included studies and were therefore organized thematically according to the main construct assessed. First, cooking or culinary skills referred primarily to practical food preparation abilities, confidence, autonomy, self-efficacy, and technical competence in preparing meals. These skills were sometimes assessed together with related food management abilities, such as meal planning, ingredient selection, storage, and leftover use. Second, some studies included broader food literacy-related constructs, encompassing food knowledge, food selection, planning, and the ability to apply nutrition or sustainability information to everyday food choices. Third, home cooking behaviors referred to enacted practices, such as frequency of cooking at home, preparation of meals from basic ingredients, and household engagement in meal preparation. Overall, exposures were harmonized into five main categories: cooking/culinary skills (n = 12), cooking/culinary education interventions (n = 5), home cooking behavior/frequency (n = 5), teaching kitchen or cooking classes (n = 4), and culinary medicine or coaching approaches (n = 2). The most frequent operationalization concerned cooking or culinary skills, generally measured as perceived confidence, ability, autonomy, or self-efficacy in performing food preparation tasks, sometimes combined with indicators of meal planning, food selection, or enjoyment of cooking [51,55,56,57,58,61,62,63,64,67,68,70]. In some studies, cooking-related skills were assessed alongside broader food literacy constructs, including food knowledge, food selection, and food preparation competencies. For example, Mengi Çelik et al. [64] examined cooking and food preparation skills together with food literacy as predictors of ultra-processed food consumption, highlighting the conceptual overlap between practical cooking abilities and broader food literacy domains.

A second group of studies assessed exposure in terms of home cooking practices or cooking frequency, typically focusing on the habitual preparation of meals at home, frequency of cooking from basic ingredients, or household engagement in meal preparation, thereby capturing cooking as an enacted behavior rather than as a perceived competence [54,59,60,65,66].

Intervention-based studies defined exposure as participation in structured cooking education, teaching kitchen, or culinary medicine/coaching programs, including online nutrition and cooking courses, workshop-based cooking classes, experiential teaching kitchens, and individualized or group culinary coaching models; in these studies, the exposure was therefore represented by program attendance and engagement rather than by a single baseline skill score [50,52,53,69,71,72,73,74,75,76,77].

Exposure assessment was predominantly based on self-reported instruments, most often administered through online surveys [50,51,52,55,56,57,58,59,62,63,64,65,66,67,68,77], whereas a smaller number of studies used face-to-face interviews [54,60,70], paper-based questionnaires [69], or mixed participatory approaches such as Photovoice and pre-post workshop evaluations [71], highlighting substantial methodological heterogeneity in how cooking-related exposures were captured across the literature.

With regard to measurement quality, 15 studies used exposures classified as validated, whereas 10 studies relied on non-validated measures, and 3 studies did not provide sufficient information to determine validation status [60,63,75]; validated approaches included standardized cooking skills instruments and a modified stage-of-change scale for home cooking, while many other studies relied on ad hoc questionnaires developed for the specific study context. Details are reported in Table 1.

### 3.4. Outcomes of Interest (Food Sustainability-Related Practices, Food Waste, Diet Quality, and Environmental Indicators)

The outcomes assessed across the included studies were heterogeneous, but could be grouped into three main domains: food waste, diet quality, and food sustainability-related practices. Food waste was the most frequently examined outcome, either as a stand-alone endpoint or in combination with dietary indicators, whereas diet quality was evaluated through measures of adherence to the Mediterranean diet, consumption of fresh or healthy foods, and intake of processed or ultra-processed foods. Only one study specifically examined food sustainability through an environmental indicator of dietary greenhouse gas emissions [56].

#### 3.4.1. Food Waste and Household Food Resource Management

Food waste outcomes were assessed in 19 studies and were operationalized using a wide range of approaches, including self-reported food waste reduction behaviors, household food waste prevention practices, awareness and confidence regarding food waste, leftover management, direct food waste audits, and qualitative exploration of preparation losses and waste-related practices [54,55,57,58,59,62,63,65,66,67,68,69,70,71,72,73,74,75,77]. Most of these studies (n = 15/19) reported a beneficial association between higher cooking skills, greater home-cooking involvement, or participation in cooking-based interventions and lower food waste or better food waste prevention behaviors [54,55,57,59,62,65,66,67,68,70,71,72,73,74,75]. However, some findings were mixed or less consistent, particularly in studies showing null findings for specific dimensions of food skills, variable changes across waste categories, or inverse associations depending on the way food waste prevention behavior was scored [58,63,69,77].

#### 3.4.2. Sustainable Diets and Diet Quality Indicators

Diet quality outcomes were assessed in 12 studies [50,51,52,53,58,60,61,64,69,71,73,76], either alone or jointly with food waste, and can be further subdivided into three subdomains: Mediterranean diet adherence (n = 4) [51,52,60,61], fresh food (fruits and/or vegetables) (n = 4) [50,53,69,71] or healthy diet indicators (n = 2) [58,76], and processed or ultra-processed food consumption (n = 6) [50,53,60,64,73,76]. Mediterranean diet adherence was evaluated using validated instruments such as MEDAS [51], MediLite [52], the alternative Mediterranean Diet score [61], or the Mediterranean Diet Score [60], and was generally positively associated with cooking skills or improved after cooking-based interventions [51,52,61], although one study reported a small non-significant association [60]. Fresh fruit or healthy diet indicators were assessed through measures such as fruit and vegetable intake, variety of fruit consumption, fish and grains/legumes consumption, and Healthy Eating Index score; overall, these studies suggested that greater cooking engagement or participation in culinary interventions was associated with healthier dietary profiles and higher consumption of fresh foods [50,53,58,69,71,76]. Processed or ultra-processed food consumption was examined through ready-meal use, ready-made food consumption, NOVA-based ultra-processed food intake, or purchases of processed food products, and findings consistently suggested that higher cooking skills or participation in culinary medicine or cooking programs were associated with lower consumption or purchasing of processed and ultra-processed foods.

#### 3.4.3. Direct Environmental Sustainability Indicators

Direct environmental sustainability indicators were rarely assessed. Only one study specifically examined diet-related greenhouse gas emissions using dietary intake data linked to the French Agribalyse database [56]. In that study, cooking skills were associated with better nutritional quality, but no clear association was observed between cooking skills and diet-related greenhouse gas emissions, suggesting that nutritional and environmental dimensions of diet may not necessarily overlap fully within this field of research [56].

Overall, several studies explored more than one outcome domain simultaneously, most commonly combining food waste with diet quality indicators, thereby highlighting the multidimensional nature of the relationship between cooking-related exposures and food sustainability practices [58,69,71,73]. More details are reported in Table 2.

### 3.5. Risk of Bias Assessment

The methodological quality of cross-sectional studies was assessed using the Newcastle–Ottawa Scale adapted for cross-sectional studies (NOS-xs) [32]. Overall, 16 cross-sectional studies were evaluated, with scores ranging from 2 to 8 stars, indicating considerable variability in methodological quality. Two studies were judged to have a low risk of bias, scoring ≥ 7 stars [56,60]. The majority of studies (n = 9) were classified as having a moderate risk of bias, with scores between 4 and 6 stars [51,55,57,58,59,61,62,64,66]. These studies generally demonstrated adequate outcome assessment but often lacked representative sampling strategies or sufficient adjustment for potential confounders. Five studies were rated as having a high risk of bias, scoring ≤ 3 stars [54,63,65,67,68]. In these studies, the main methodological limitations included limited representativeness of the study population, inadequate control of confounding variables, and reliance on self-reported exposure and outcome measures. Across studies, the NOS-xs domains most frequently unmet were related to sample representativeness and adjustment for confounders, whereas outcome measurement and statistical analysis were more consistently addressed.

Eight studies employing pre–post quasi-experimental designs were appraised using the Joanna Briggs Institute (JBI) Critical Appraisal Checklist for Quasi-Experimental Studies. Overall methodological quality ranged from moderate to low risk of bias. Four studies were classified as having a low risk of bias [52,73,75,76], while four studies were judged to have a moderate risk of bias [50,71,72,74]. The most common methodological limitation across studies was the absence of a control group, which is typical of single-group pre–post intervention designs. Nevertheless, several studies demonstrated strengths, including repeated outcome measurements before and after the intervention and the use of appropriate statistical analyses. Some studies also presented limitations related to incomplete reporting of follow-up procedures or limited consideration of potential confounding factors.

Two qualitative studies were assessed using the JBI Critical Appraisal Checklist for Qualitative Research. Both studies [53,70] were judged to have moderate methodological quality. Overall, the studies demonstrated appropriate qualitative methodologies, clear research aims, and adequate data collection and analysis procedures. However, limited reporting of researcher reflexivity and the influence of the researcher on the research process was observed.

The cluster randomized pilot trial by Metcalfe et al. [69] was assessed using the Cochrane RoB 2 tool for cluster-randomized trials [78] and was judged to present some concerns of bias, mainly due to limited information on the randomization process, potential recruitment bias within clusters, and reliance on self-reported outcome measures.

The overall risk of bias judgment is reported in Table 2. A detailed summary of the risk-of-bias assessment for all included studies is presented in Supplementary Table S3.

## 4. Discussion

The present systematic review provides a comprehensive synthesis of the available evidence on the relationship between cooking-related skills and food sustainability practices. Overall, the evidence base is characterized by considerable methodological and clinical heterogeneity, which should be taken into account when interpreting the findings. Studies were conducted across a wide range of countries, settings, and population groups, but the overall quality of the evidence varied, with the majority of cross-sectional studies judged to have a moderate risk of bias, and only a limited number classified as having a low risk of bias. In addition, the predominance of cross-sectional designs, together with substantial variation in sample sizes and target populations, limits the ability to draw causal inferences. The exposure domain was also conceptually broad and multidimensional, encompassing cooking skills, home cooking behaviors, and participation in cooking-oriented educational or coaching interventions, which further complicates direct comparison between studies. Generational differences may represent an important contextual factor in the relationship between cooking-related skills and food sustainability-related practices. The included studies involved heterogeneous adult populations, ranging from university students and young adults to middle-aged and older community samples. Younger adults may face specific barriers to home cooking, including limited time, lower cooking confidence, greater reliance on convenience foods or food delivery services, and reduced intergenerational transmission of culinary knowledge. Conversely, older adults may have greater familiarity with traditional food preparation practices, leftover use, and household food resource management, although these patterns may vary according to socioeconomic, cultural, and household contexts. However, most included studies did not report age-stratified or generation-specific analyses. Therefore, potential generational differences should be considered as an important interpretative issue and a priority for future research, rather than as a firm conclusion of the present review.

Despite these limitations, the available evidence suggests preliminary associations between cooking skills and related practices are more favorable food sustainability-related behaviors, particularly reduced food waste and improvements in diet quality, including greater adherence to healthy dietary patterns. However, these findings should be interpreted cautiously, as much of the evidence comes from cross-sectional studies, studies using self-reported measures, or studies with moderate to high risk of bias. Evidence linking these exposures to broader environmental sustainability indicators remains comparatively limited. Taken together, these findings highlight both the potential relevance of cooking-related competencies for promoting more sustainable food behaviors and the need for more robust and methodologically rigorous studies to strengthen the evidence base.

### 4.1. Hypothesized Pathways Linking Cooking Skills and Sustainable Food Behaviors

The observed association between cooking skills and sustainable food behaviors may be interpreted by several plausible, although not yet fully established, pathways. First, individuals with higher cooking skills may be more inclined or better equipped to prepare meals from scratch, using fresh foods and minimally processed ingredients, avoiding or highly reducing pre-packaged and ultra-processed foods, which are associated with greater environmental footprints [79,80]. Second, cooking skills are closely associated with food literacy [81] which in turn facilitates the selection of more sustainable ingredients such as seasonal, locally sourced, and plant-origin foods [82]. An important conceptual issue concerns the extent to which cooking skills act independently or as part of the broader construct of food literacy. Food literacy includes not only practical food preparation skills, but also knowledge, planning, food selection, budgeting, label interpretation, and the ability to apply nutrition and sustainability information in everyday food choices. From this perspective, cooking skills may represent the practical and behavioral component through which broader food literacy is translated into action. For example, the ability to recognize ultra-processed foods, plan meals, purchase appropriate ingredients, store food correctly, and use leftovers may be as important as technical cooking competence itself. Therefore, the associations observed in this review should not be interpreted as evidence that cooking skills alone determine sustainable food practices, but rather that cooking-related competencies may operate within a wider food literacy framework.

Third, higher cooking-related skills may be accompanied by greater ability in meal planning and ingredient management, which are associated with reduced household food waste, one of the most impactful contributors to environmental sustainability [83]. Fourth, cooking confidence may lower the perceived barriers to adopting plant-based dietary patterns, which are consistently associated with lower greenhouse gas emissions, reduced land and water use, and greater biodiversity preservation compared to animal-based diets [84,85,86,87]. Finally, culinary skills may operate through social and cultural pathways: individuals who cook regularly are more likely to transmit food knowledge and sustainable practices within family and community networks, thereby amplifying individual behaviors into collective dietary change [88,89]. Taken together, these hypothesized pathways suggest that cooking skills may function not merely as a practical competence, but as a broader enabler of health-promoting and environmentally responsible food behaviors, consistent with both One Health and Planetary Health frameworks.

### 4.2. Strengths and Limitations

This systematic review has several strengths. First, the review was conducted following a rigorous and transparent methodology, including a predefined protocol, comprehensive database searches, and study selection and data extraction performed by independent reviewers. In addition, the methodological quality of the included studies was assessed using design-specific appraisal tools, which allowed a more appropriate evaluation of potential sources of bias across heterogeneous study designs. However, several limitations should be acknowledged. The available evidence was characterized by substantial methodological heterogeneity, including differences in study design, exposure definitions, outcome assessment methods, and study populations, which limited the feasibility of quantitative synthesis. Moreover, the predominance of cross-sectional designs restricts the ability to draw causal inferences, while several studies relied on self-reported measures of cooking behaviors and sustainability outcomes, potentially introducing measurement bias. Another important limitation concerns the sex distribution of the included populations. Women were overrepresented across the evidence base, with several studies including predominantly female samples and some studies conducted entirely or almost entirely among women. This pattern may reflect the fact that many studies recruited household food shoppers, meal providers, or individuals responsible for domestic food preparation, roles that remain strongly gendered in many social and cultural contexts. However, this imbalance may limit the applicability of the findings to men and to households where food-related responsibilities are differently distributed. Since cooking-related skills, meal planning, food purchasing, and food waste management may be influenced by gender norms and household roles, the associations observed in the included studies cannot be assumed to apply equally to men. Moreover, the lack of sex-stratified analyses in most studies prevented us from assessing whether sex modifies the relationship between cooking-related skills and food sustainability practices. Another limitation is that the review was restricted to peer-reviewed scientific articles and did not include grey literature, such as technical reports from public health agencies, government nutrition program evaluations, or documents from international organizations. This may have led to the exclusion of relevant implementation evidence, particularly for teaching kitchen and culinary medicine programs that are often developed or evaluated within public health practice settings but not necessarily published in peer-reviewed journals. Therefore, the findings of this review should be interpreted as reflecting the peer-reviewed evidence base, while real-world implementation experiences may be underrepresented. Finally, the limited number of studies evaluating broader environmental sustainability indicators highlights an important gap in the current evidence base.

### 4.3. Future Directions

Future research should aim to strengthen the current evidence base by addressing the methodological limitations identified in this review. In particular, there is a clear need for well-designed longitudinal and experimental studies, including randomized or quasi-experimental interventions, to better clarify the causal relationship between cooking-related skills and food sustainability outcomes. Many of the existing studies relied on cross-sectional designs and self-reported measures, which limit the ability to infer causality and increase the risk of measurement bias. Future studies should therefore employ standardized and validated instruments to assess cooking skills, dietary behaviors, and sustainability-related outcomes, and should incorporate objective indicators, such as direct measurement of food waste or environmental impact metrics. In addition, greater conceptual consistency in the definition and measurement of cooking-related exposures would improve comparability across studies. Indeed, the current evidence does not allow the independent contribution of cooking skills and food literacy to be clearly disentangled. Only a limited number of studies measured both constructs simultaneously, and most available studies used cross-sectional designs, which limits causal interpretation. Future studies should therefore examine whether cooking skills mediate, moderate, or independently contribute to the relationship between food literacy and sustainable food practices.

Research should also expand beyond diet quality and food waste to include broader environmental sustainability indicators, such as greenhouse gas emissions, resource use, and sustainable food procurement practices. Finally, large-scale studies conducted in diverse populations and settings, with adequate adjustment for potential confounding factors, are needed to provide more robust and generalizable evidence on the role of cooking-related competencies in promoting sustainable food behaviors. Future studies should aim to recruit more sex-balanced samples and report sex-stratified analyses, in order to clarify whether the relationship between cooking-related skills and food sustainability practices differs between women and men.

### 4.4. Public Health Implications

The findings of this review have important implications for public health, particularly in the context of the growing challenges posed by climate change, food security, and sustainable food systems. Cooking-related skills may represent a relevant and modifiable behavioral pathway through which individuals can adopt more sustainable dietary practices, including reduced food waste and improved diet quality. Strengthening cooking competencies could potentially contribute not only to healthier eating patterns but also to more efficient food resource use and reduced environmental pressure on food and health systems [90]. From a public health practice perspective, integrating cooking education and practical food skills into health promotion programs, community interventions, and educational curricula may represent a promising strategy to support sustainable dietary transitions [91,92,93]. However, cooking education should ideally be embedded within broader food literacy interventions, addressing not only technical cooking abilities but also meal planning, food selection, budgeting, label reading, recognition and reduction of ultra-processed foods, sustainable procurement, safe food storage, and food waste prevention. This broader approach may be more effective in supporting sustainable food practices than interventions focused exclusively on cooking techniques. In addition, improving cooking literacy may enhance individuals’ ability to manage food safely, plan meals effectively, and reduce food loss at the household level, thereby contributing to both food safety and food security [94].

From a policy perspective, the findings support the inclusion of cooking and food literacy components in pilot health promotion, community nutrition, university, workplace, and sustainability programs, provided that these initiatives include clear objectives, standardized outcome measures, equity considerations, and built-in evaluation frameworks. At present, however, the evidence does not support cooking skills as a stand-alone policy solution, but rather as one potentially useful component of broader strategies aimed at promoting healthier and more sustainable food behaviors. Future policies and programs should therefore combine practical food skills with broader actions addressing food environments, affordability, access to fresh and minimally processed foods, and household food waste reduction. Given the increasing need to develop resilient and sustainable food systems in the face of climate [95] change, policies and interventions that promote cooking skills could play a supportive role in advancing co-benefits for human health, environmental sustainability, and food system resilience.

## 5. Conclusions

Overall, the available evidence suggests that cooking-related skills and home cooking practices may be associated with more selected food sustainability-related behaviors, particularly through reduced food waste and improvements in diet quality. The evidence appears relatively more consistent for food waste reduction and household food resource management, as well as for selected diet quality indicators, including greater adherence to healthy dietary patterns, higher consumption of fresh or minimally processed foods, and lower consumption of processed or ultra-processed foods.

From a practice perspective, these findings suggest that nutrition education, teaching kitchen, culinary medicine, and community-based food programs should not focus exclusively on technical cooking abilities but should integrate cooking practice within broader food literacy approaches. Such programs should address meal planning, food selection, budgeting, recognition and reduction of ultra-processed foods, safe food storage, leftover use, and household food waste prevention. For policymakers, the findings support the inclusion of cooking and food literacy components in pilot health promotion and sustainability programs, provided that these initiatives include built-in evaluation frameworks and do not assume effectiveness without further evidence.

Moreover, the current evidence base is characterized by substantial methodological heterogeneity and is largely dominated by cross-sectional studies, overrepresentation of women and primary household food preparers, limiting the ability to draw causal conclusions and limiting the generalizability to men. Therefore, the evidence should be considered preliminary and hypothesis-generating rather than confirmatory. More high-quality longitudinal and experimental research using standardized and validated measures of both cooking skills and sustainability-related practices is needed to strengthen the evidence base. Strengthening cooking competencies, particularly when embedded within broader food literacy approaches, may represent a promising strategy to support healthier and more sustainable food systems in the context of climate change and growing food security challenges.

## Figures and Tables

**Table 1:** Descriptive characteristics of included studies, reported in alphabetical order.

Author, Year [Ref]	Country	Study Period	Study Design	Unit	Final Sample Size	Attrition n° (%)	Women %	Age	Population Characteristics	Type of Exposure	Exposure Assessment	Exposure Assessed Using Validated Methods
Abeliotis, 2016 [54]	Greece	2012	Cross-sectional	Household/respondent	231	24 (9.4)	NA	NA	Adults/general population	Home cooking behavior/frequency	face-to-face interview	No
Adam M., 2015 [50]	USA	January–February 2014 (5-week course)	Pre-post intervention	Individual	7422	14,601 (66.3)	86.4	<20 3.2%; 20–29 28.3%; 30–39 41.1%; 40–49 17.7%; ≥50 9.8%	Adults enrolled in online course, mostly women, child-rearing	Cooking/culinary education intervention	online survey	No
Aloysius N., 2025 [55]	Australia	4–19 December 2023	Cross-sectional	Household	1007	NA	50.9%	18–24 9.4%; 25–44 37.5%; 45–64 35.7%; ≥65 17.3%	Adults/nationally representative households; majority responsible for household food management	Cooking/culinary skills	online survey	Yes
Arrazat L., 2024 [56]	France	March–April 2022	Cross-sectional	Individual	582	211 (26.6)	56%	21.0 ± 2.6 years	University students	Cooking/culinary skills	online survey	Yes
Bender K.E., 2022 [57]	USA	June–July 2020 (COVID-19 pandemic)	Cross-sectional	Household	946	NA	76%	45.6 ± 16.4 years	Adults/household food shoppers responsible for cooking	Cooking/culinary skills	online survey	Yes
Burrington C.M., 2020 [71]	USA	Summer 2018 (12 weeks)	Pre-post intervention	Family/household	19	10 (34.5)	93%	NR	Low-income rural families with ≥1 child; majority mothers.	Cooking/culinary education intervention	program participation with self-reported surveys and Photovoice activities	No
Carroll N., 2021 [58]	Canada	February–August 2017	Cross-sectional	Household	85	NA	82%	36.5 ± 4.5 years	Parents of young children aged 2–8 years, mostly White, highly educated	Cooking/culinary skills	online survey	Yes
Casucci M., 2026 [51]	Spain	May–December 2024 (data collection period)	Cross-sectional	Individual	233	55 (19.1)	74.1%	55.0 ± 13.0 years	Adults/general population recruited via convenience sampling in structured community settings.	Cooking/culinary skills	online survey	Yes
Cemali O., 2025 [72]	Turkey	25 April 2025 (single-day workshop held during the 14th International Trakya Family Medicine Congress)	Pre-post intervention	Individual	17	8 (32.0)	Predominantly female; 24/25 women at enrolment	Adult family physicians; exact age NR	Family physicians	Teaching kitchen/cooking classes	Pre- and post-workshop quantitative questionnaires (Likert-scale self-report)	No
Chen H., 2019 [59]	USA	NA	Cross-sectional	Household	515	NA	53%	Majority 26–55 years	Adults/primary meal providers	Home cooking behavior/frequency	online survey	Yes
Di Costanzo, 2025 [60]	Italy	2005–2010	Cross-sectional	Individual	11,982	913 (7.1)	Women only	55.0 ± 11.7 years	Adult women/population cohort	Home cooking behavior/frequency	face-to-face interview	NA
Finkelstein, 2025 [53]	USA; Israel	Qualitative data collected between December 2019 and September 2022; perceptions captured at end of 3-month intervention, 6 months, and 12 months from intervention initiation.	Qualitative	Individual	24	15 (38.5)	17/24 women	44.17 ± 12.92 years	Adults with overweight/obesity	Culinary medicine/coaching	Validated modified University of Rhode Island Change Assessment (URICA) scale at baseline for stage of change regarding home cooking; participation in a 3-month culinary coaching telemedicine intervention (12 weekly one-on-one 30 min tele-sessions with a culinary coach).	Yes
Garcia A.L., 2017 [73]	United Kingdom (Scotland)	2015–2016	Pre-post intervention	Individual/household cook	88	29 (24.8)	80%	45.6 ± 13.1 years	Adults from disadvantaged communities	Cooking/culinary education intervention	Self-reported questionnaires (food practices, shopping, confidence, cooking frequency) administered pre- and post-intervention	No
Garcia T., 2021 [74]	USA	NA	Pre-post intervention	Individual	45	NA	84%	48.0 ± 15.8 years	Community health center clients	Teaching kitchen/cooking classes	other, specify	No
Gonçalves, 2025 [61]	Portugal	June 2019 to January 2021;	Cross-sectional	Individual	111	3 (2.6)	54.1%	47.6 ± 10.5 years	University staff/adults attending annual mandatory occupational health appointments	Cooking/culinary skills	Self-reported Cooking Skills Scale (CSS)	Yes
Karunasena G.G., 2021 [62]	Australia	2019–2020	Cross-sectional	Household	2187	3585 (62.1)	57.8%	18–24 6.2%; 25–44 43.1%; 45–64 33.1%; >65 17.6%	Adults/household food managers	Cooking/culinary skills	online survey	No
Laila A., 2024 [75]	Canada	October–November 2020	Pre-post intervention	Family/household	17	2 (10.5)	55% (family sample)	Children mean age 10.1 years; parent age NR	Families with children	Cooking/culinary education intervention	survey	NA
Marconi S., 2026 [52]	Italy	March 2025	Pre-post intervention	Individual	28	14 (33.3)	67.9%	25.2 ± 5.5 years	University students (University of Brescia), living away from home	Teaching kitchen/cooking classes	online survey	Yes
Meixner O., 2020 [63]	Austria	2017	Cross-sectional	Household	470	0	74.3%	Age groups only (≤20; 21–30; 31–45; 46–60; ≥61)	Adults/general population (mostly women), living in urban area	Cooking/culinary skills	online survey	NA
Mengi Çelik, 2025 [64]	Turkey	January 2025–March 2025	Cross-sectional	Individual	3572	NA	67.6%	33.7 ± 13.5 years	Adults/general population	Cooking/culinary skills	online survey	Yes
Metcalfe J.J., 2022 [69]	USA	June–September 2018	Cluster randomized pilot trial	Individual/household	120	22 (15.5)	72% full sample (82% PAE; 75% EO; 60% control)	40 ± 12 years full sample	Low-income adults/family meal preparers	Teaching kitchen/cooking classes	paper-based survey	Yes
Morosan E., 2024 [65]	Romania	15 April 2023 to 15 May 2023	Cross-sectional	Household	300	NA	81.67%	Age groups only	Adults/general population	Home cooking behavior/frequency	online survey	Yes
Nonomura M., 2019 [70]	Japan	2017–2018	Qualitative	Individual household cook	9	0	100% women	20–70 years	Women responsible for household cooking	Cooking/culinary skills	face-to-face interview	No
Polak R., 2018 [76]	Israel	2012–2013	Pre-post intervention	Community	490		49%	NA	Community residents (kibbutz)	Culinary medicine/coaching	Participation in 8 group coaching sessions and culinary training involving kitchen staff, preschool staff, and residents, implemented through a community-based participatory approach (CBPA)	No
Remolina I., 2025 [77]	USA	2024	Pre-post intervention	Individual	36	39	90% intervention; 75% control	20.5 ± 1.48 intervention; 20.0 ± 1.40 control	University students, mostly Asian	Cooking/culinary education intervention	online survey	Yes
Rodgers R.F., 2021 [66]	USA	2020	Cross-sectional	Individual	954		79% USA.; 78% Italy	30.5 ± 10.9 U.S.; 33.8 ± 12.9 Italy	Adults/general population	Home cooking behavior/frequency	online survey	No
Romani S., 2018 [67]	Italy	2016–2017	Cross-sectional	Household	210	48	64.6% (Study 1); 69.5% (Study 2); 56.9% (Study 3)	Multi-study age distributions reported separately	Adults responsible for shopping/cooking. Households with at least one child	Cooking/culinary skills	online survey	Yes
Yetkin Özbük R.M., 2022 [68]	Turkey	2021	Cross-sectional	Household	511		75.5%	29 or younger 14.7%; 30–40 34.4%; 41–51 31.1%; ≥52 19.8%	Adults responsible for shopping/cooking	Cooking/culinary skills	online survey	Yes

EO: educational classes only; NA: not available; PAE: produce allocations with educational classes; USA: United States of America; ° attrition = number of participants lost to follow-up or excluded from the initial sample size.

**Table 2:** Characteristics of the included studies and summary of main findings assessing the association between cooking skills, home cooking, or cooking-based interventions and selected food sustainability-related outcomes, grouped into food waste and household food resource management, diet quality (Mediterranean diet adherence, fresh food consumption, and processed/ultra-processed food intake), and environmental indicators.

Author, Year [Ref]	Outcome of Interest	Outcome Assessment	Direction of the Effect	Main Results	Adjustment Model	CoI	Funds	Risk of Bias
Abeliotis K., 2016 [54]	Food waste	ad hoc	Beneficial	Higher vs. lowest cooking group mean 3.37 vs. 2.36 on 0–6 scale	No	No	Yes	High
Adam M., 2015 [50]	Diet quality (processed and fresh food)	Online pre- and post-course self-report surveys (Likert scales, categorical variables)	Beneficial	Fresh-food home cooking 63.4% → 71.4% (*p* < 0.0001); dinners cooked at home with fresh foods increased (66.1% → 72.1%, *p* < 0.0001); fresh vegetables at dinner (71.4% → 77.3%, *p* < 0.0001); fresh fruit at dinner (28.4% → 34.2%, *p* < 0.0001); red meat decreased (30.3% → 26.7%, *p* < 0.0001); perception of dinner as very/extremely healthy (39.3% → 56.4%, *p* < 0.0001) and enjoyable (55.2% → 66.7%, *p* < 0.0001).	Analyses stratified by gender, age, education, perceived weight, USA vs. non-USA	No	Yes	Moderate
Aloysius N., 2025 [55]	Food waste	Self-reported survey on 5 types of leftovers (prepared-not plated, plated-not eaten, leftover ingredients, takeaway leftovers, online food order leftovers), 7-point Likert scale	Beneficial	better ability higher (β = 0.499) to leftover management; better leftover management (β = −0.272) reduced food waste. All *p* < 0.001.	SEM (no confounder adjustment stated)	No	Yes	Moderate
Arrazat L., 2024 [56]	Food sustainability	GHGE estimated from dietary intake using the French Agribalyse database applied to validated FFQ data.	Null	β = −0.02 (95% CI −0.18 to 0.15) for diet GHGE	Models adjusted for age, gender, scholarship status, living conditions + 14 behavioral determinants.	No	Yes	Low
Bender K.E., 2022 [57]	Food waste	online survey food waste attitudes and behaviors self-reported	Beneficial	OR = 0.87 (95% CI 0.82–0.93) for cooking skills and household food waste; food management skills (OR = 0.94, 95% CI 0.90–0.98), and more frequent home cooking (OR = 0.90, 95% CI 0.84–0.97) were associated with reduced household food waste	Logistic regression adjusted for age, sex, race/ethnicity, education, income, food security status	No	Yes	Moderate
Burrington C.M., 2020 [71]	Food waste and diet quality (fresh food)	Pre- and post-intervention self-administered surveys; Photovoice qualitative feedback	Beneficial	Fruit variety consumed increased (*p* = 0.01) and vegetable consumption increased (*p* = 0.05); families reported improved meal planning, more home cooking, children eating more FV, and reduced waste due to better planning and storage (reported qualitatively).	No	No	Yes	Moderate
Carroll N., 2021 [58]	Food waste and diet quality	Diet quality: 3-day food records (parents) scored with Healthy Eating Index-2015; Food waste: direct household waste audit (7 days, separation of food waste categories)	Mixed/unclear. Beneficial for diet quality	Mechanical food skills β = −0.25 (*p* = 0.03) for unavoidable waste; total skills null on food waste. Higher food skills associated with better diet quality (β = 0.27, *p* = 0.01)	Models adjusted for age, sex, household income, education, marital status, BMI.	No	Yes	Moderate
Casucci M., 2026 [51]	Diet quality (MD adherence)	Mediterranean Diet Adherence Screener (MEDAS)	Beneficial	Cooking skills (FCSk) were significantly positively associated with MEDAS score: Standardized β = 0.189 (*p* = 0.001) for cooking skills and MEDAS	No adjustment variables applied in the main regression models. All reported models are univariable (unadjusted) OLS regressions. Authors explicitly state that multivariable adjustment was not applied because sociodemographic/socioeconomic indicators were not measured in a fully harmonized, comparable way across age groups (e.g., parental education for minors vs. individual education for adults). Potential confounders (sex, age, SES, parental education, employment) were collected and described but not included in regression models. Authors acknowledge this as a limitation.	No	Yes	Moderate
Cemali O., 2025 [72]	Food waste	pre- and post-workshop Likert scores	Beneficial	Food-waste reduction awareness Z = −3.078 (*p* = 0.002).	No adjustment variables applied (pre-post within-subject design using non-parametric Wilcoxon test; no multivariate adjustment for confounders due to single-arm pilot design and small sample size)	No	No	Moderate
Chen H., 2019 [59]	Food waste	Online self-report survey	Beneficial	β = 0.18 (*p* < 0.01) for home cooking and food waste prevention behavior	Multivariable regression adjusted for demographic variables (gender, age, education, income) and behavioral constructs (environmental concern, awareness, buying impulsiveness, eating out).	No	No	Moderate
Di Costanzo G., 2025 [60]	Diet quality (MD adherence and processed food consumption)	MDS by Trichopoulou et al., and NOVA classification	Null for MD adherence Beneficial for UPF	MDS β = 0.07 (95% CI −0.03 to 0.17); UPF β = −0.16 (95% CI −0.26 to −0.07)	Model 2 adjusted for age, energy intake, educational level, housing tenure, place of residence, marital status, occupational class, smoking status, BMI, leisure-time physical activity, history of cardiovascular disease, history of cancer, diabetes, hypertension, and hyperlipidemia.	No	Yes	Low
Finkelstein A., 2025 [53]	Diet quality (processed and fresh food)	Qualitative open-ended questionnaires and semi-structured Zoom interviews	Beneficial	Qualitative reports of more fresh ingredients/home cooking and less ready-made foods	NA	Yes	Yes	Moderate
Garcia A.L., 2017 [73]	Food waste and diet quality (processed and fresh food)	Pre- and post-intervention self-administered questionnaires	Beneficial	Ready meal use 60% → 38%; Cooking from scratch increased from 59% to 82% (*p* < 0.001); 65% reported less food waste	No	No	Yes	Low
Garcia T., 2021 [74]	Food waste	ad hoc	Beneficial	Confident about food waste (98% yes), Improved knowing how to use food before spoilage (*p* = 0.017)	two-sided paired *t* tests	No	Yes	Moderate
Gonçalves, 2025 [61]	Diet quality (MD adherence)	Validated semiquantitative Food Frequency Questionnaire (FFQ) covering the previous 12 months was used to derive the alternative Mediterranean Diet (aMED) score; one 24 h dietary recall was used to classify foods by NOVA processing level.	Beneficial	higher cooking skills were positively associated with Mediterranean diet. Adjusted β = 0.299 (*p* = 0.003) for cooking skills and aMED	Age; number of persons in household	No	Yes	Moderate
Karunasena G.G., 2021 [62]	Food waste	ad hoc	Beneficial	Difficulty in leftover cooking associated with food waste (χ^2^ = 153.5, *p* < 0.001)	No clear confounder adjustment	No	No	Moderate
Laila A., 2024 [75]	Food waste	audits at baseline and postintervention	Beneficial	Avoidable fruit/veg waste −0.11 kg/week (*p* = 0.02)	No	NA	Yes	Low
Marconi S., 2026 [52]	Diet quality (MD adherence)	MediLite questionnaire (validated): assesses consumption of 9 food groups (fruits, vegetables, cereals, legumes, fish, meat, dairy, alcohol, olive oil); total score 0–18; categories: low (≤8), moderate (9–11), high (≥12)	Beneficial	MediLite score 9.43 → 10.9 (*p* = 0.006)	No	No	No	Low
Meixner O., 2020 [63]	Food waste	online survey and face-to-face survey	Detrimental	Cooking skills vs. food-waste prevention behavior r = −0.549 (*p* ≤ 0.001)	No	NA	NA	High
Mengi Çelik O., 2025 [64]	Diet quality (processed food)	Screening Questionnaire of Highly Processed Food Consumption (11 items; score 0–11; ≥6 indicates high consumption)	Beneficial	Higher UPF consumption was inversely associated with cooking and food preparation skills and food literacy. Standardized β = −0.095 [95% CI 0.003 to 0.008] (*p* < 0.001) for cooking skills and UPF score	Gender, age, Food Literacy Tool total score, Cooking and Food Preparation Skills Scale total score	No	No	Moderate
Metcalfe J.J., 2022 [69]	Food waste and diet quality (fresh food)	structured interviews	Mixed/unclear	About half reported less waste/waste-reduction behaviors; some allocated produce discarded. Greater improvement in “serving vegetables to the family” pre–post in PAE vs. control (*p* = 0.010).	Multilevel linear regression models (continuous outcomes) All models are adjusted for the following covariates: Seasonality: month when post-program data were collected (class/cohort-level variable) Gender Age Race Ethnicity M2MP program completion Distance from home to program site Number of children Monthly food budget Participation in food assistance programs (e.g., SNAP/WIC) Multilevel logistic regression model (binary outcome): In addition to all covariates listed above, the logistic model also adjusts for: Baseline (pre-intervention) farmers’ market shopping response	No	Yes	Some concerns
Morosan E., 2024 [65]	Food waste	On-line survey	Beneficial	Greater home cooking involvement more common in low-food-waste groups. 75% of the FW-0 group are very implied in Home Cooking and 68.89% of FW-3 prefer to cook 2–3 times a week.	No	Yes	Yes	High
Nonomura M., 2019 [70]	Food waste	face-to-face interview + observation (coding categories of waste: avoidable, excessive, unintentional)	Beneficial	Qualitative evidence that lack of skill/knowledge contributed to preparation losses. Preparation losses were not only cultural but also linked to culinary skills and knowledge gaps	NA	No	Yes	Moderate
Polak R., 2018 [76]	Diet quality (processed and fresh food)	Food procurement management software combined with qualitative focus groups	Beneficial	Fish purchases +115% (*p* < 0.001); whole grains/legumes +77% (*p* < 0.001); whole wheat bread +1381% (*p* < 0.001); processed meats −55% (*p* < 0.001); margarine −100% (*p* < 0.001); industrial sauces −38% (*p* < 0.05)	Analysis using mixed linear models with FDR correction for multiple comparisons (Benjamini–Hochberg)	No	Yes	Low
Remolina I., 2025 [77]	Food waste	online survey (U.S. Adult Food Security Survey Module; IFIC Food Waste survey)	Mixed/unclear	More food-waste awareness; some home leftover waste ↑, restaurant/dairy waste ↓	No	No	Yes	Moderate
Rodgers R.F., 2021 [66]	Food waste	online survey	Beneficial	49% overall reported decreased food waste since pandemic start; OR = 1.35 (95% CI 1.13–1.60) for more frequent cooking and decreased food waste; USA respondents more likely than Italian to report decreased food waste (OR = 0.47, *p* < 0.001 for Italy vs. USA.)	age, gender, financial insecurity, country, time since restrictions	No	No	Moderate
Romani S., 2018 [67]	Food waste	online survey	Beneficial	ANCOVA intervention effect F = 11.0, *p* < 0.001; household food waste ~1554 g pre-test to 816 g post-test	Age, gender, household size, number of children	No	Yes	High
Yetkin Özbük R.M., 2022 [68]	Food waste	online survey	Beneficial	High-skill clusters reported less food waste than low-skill cluster. Cluster 1—Careless planners and cooks (n = 90, 17.61%; low cooking skills M = 3.34, SD = 0.54; no food waste 21.1%). Cluster 2—Resourceful planners and cooks (n = 285, 55.77%; highest cooking skills M = 4.39, SD = 0.38, no food waste 38.9%). Cluster 3—Careless planners and resourceful cooks (n = 136, 26.62%; high cooking skills M = 4.33, SD = 0.41, no FW 43.4%)	segmentation controlled for demographics, health orientation, price consciousness, environmental concern	No	No	High

ANCOVA: analysis of covariance; aMED: alternative mediterranean diet score: BMI: body mass index; CI: confidence interval; FFQ: food frequencies questionnaire; FCSk: food and cooking skills; FDR: false discovery rate; FW: food waste; GHGE: greenhouse gasses emission; IFIC: international food information council; NA: not available: M2MP: Market to MyPlate; M: mean; MD: mediterranean diet; MDS: mediterranean diet score: MEDAS: mediterranean diet adherence score; MediLite: Mediterranean Diet Literature-based Adherence score; OLS: ordinary least squares; OR: odds ratio PAE: produce allocations with educational classes; SES: socioeconomic status; SD: standard deviation; SNAP: supplemental nutrition assistance program; UPF: ultra-processed food; USA: United States of America; WIC: special supplemental nutrition program for women, infants, and children.

## Data Availability

No new data were created or analyzed in this study. Data sharing is not applicable to this article.

## Supplementary Materials

The following supporting information can be downloaded at: https://www.mdpi.com/article/10.3390/foods15111899/s1, Table S1: Search string for each database; Table S2: Detailed inclusion/exclusion criteria, defined according to PECOS (Population, exposure, comparison, outcome, study design; Table S3: Risk of bias for each included study, reporting item-by-item assessment. Studies are listed in alphabetical order and stratified by study design.
