# Supplementary material for: Cooking-Related Skills and Food Sustainability-Related Practices: A Systematic Review
Source: Foods. 2026 May 28;15(11):1899. doi: 10.3390/foods15111899 (PMC13257166; doi:10.3390/foods15111899)
Supplement: Supplementary file 1 [file foods-15-01899-s001.zip › foods-4339297-supplementary.pdf]

Supplementary Table S1. Search string for each database.

Database	Search string	Number of identify records on 25 February 2026
PubMed/MEDLINE	((("Cooking"[Title/Abstract] OR "culinary"[Title/Abstract] OR "Cooking"[MeSH Terms]) AND ("education"[Title/Abstract] OR "Skill"[Title/Abstract] OR "skills"[Title/Abstract] OR "home"[Title/Abstract] OR "Nutritional Sciences"[MeSH Terms] OR "Medicine"[MeSH Terms] OR "Medicine"[Title/Abstract])) OR "teaching kitchen"[Title/Abstract]) AND ("food sustainability"[Title/Abstract] OR "sustainable diet"[Title/Abstract] OR "sustainable food"[Title/Abstract] OR "food waste"[Title/Abstract] OR "environmental impact of food"[Title/Abstract] OR "Sustainable Development"[MeSH Terms] OR "Food Loss and Waste"[MeSH Terms] OR "diet, mediterranean"[MeSH Terms] OR "food, organic"[MeSH Terms] OR "food, processed"[MeSH Terms] OR "Processed Food"[Title/Abstract] OR "organic food"[Title/Abstract] OR "seasonal food"[Title/Abstract])	215
Scopus	ABS-KEY ("teaching kitchen")) OR ((TITLE-ABS-KEY (cooking OR culinary) AND TITLE-ABS-KEY (education OR skill OR home OR nutrition OR medicine)))) AND (TITLE-ABS-KEY (("sustainable diet" OR "sustainable food" OR "food waste" OR "environmental impact of food" OR "food loss" OR "Mediterranean diet" OR "processed food" OR "organic food" OR "seasonal food")))	1082
EMBASE	ti,ab,kw OR 'culinary':ti,ab,kw) AND ('education':ti,ab,kw OR 'skill':ti,ab,kw OR 'home':ti,ab,kw OR 'nutrition':ti,ab,kw OR 'medicine':ti,ab,kw)) OR (('culinary medicine'/exp OR 'culinary medicine':ti,ab,kw) OR ('teaching kitchen':ti,ab,kw)) AND ('food sustainability':ti,ab,kw OR 'sustainable diet':ti,ab,kw OR 'sustainable food':ti,ab,kw OR 'environmental impact of food':ti,ab,kw OR 'sustainable development':ti,ab,kw OR 'food loss':ti,ab,kw OR 'food waste':ti,ab,kw OR 'mediterranean diet':ti,ab,kw OR 'processed food':ti,ab,kw OR 'organic food':ti,ab,kw OR 'seasonal food':ti,ab,kw)	464

Supplementary Table S2: Detailed inclusion/exclusion criteria, defined according to PECOS (Population, exposure, comparison, outcome, study design).

Details	Inclusion criteria	Exclusion criteria
**Population**	General adult populations (≥18 years), both women and men	No general population.<br>Subjects below 18 years old.<br>Studies in which the adult population could not be clearly separated from younger participants
**Intervention/Exposure**	Studies evaluating cooking-related skills, home cooking behaviors, culinary education, culinary medicine interventions, or teaching kitchen programs. Both validated and non-validated instruments were considered, with validation status recorded.	Studies that did not evaluate cooking-related skills, home cooking behaviors, culinary education, culinary medicine interventions, or teaching kitchen programs.<br>Studies focusing exclusively on nutrition knowledge, food literacy, or dietary education without a cooking component
**Comparison**	Different levels or categories of cooking skills (e.g., high vs. low, trained vs. untrained individuals), or, when applicable, absence of cooking skills exposure.	Other type of comparisons
**Outcome**	Food sustainability practices (including food sustainability, food waste/lost, organic food, seasonal food, adherence to Mediterranean Diet, processed food): self-reported or objectively assessed.	Outcomes unrelated to sustainability or dietary behaviors
**Study design**	Original, peer-reviewed qualitative and quantitative observational studies, or interventional studies.	Studies not published in peer-reviewed scientific journals.<br>Editorials, commentaries, narrative reviews, conference abstracts without full data, protocols, or opinion papers, or if they did not report original empirical data
**Time frame**	No filter	
**Language**	English	Other than English
**Setting**	Any geographical setting	

Supplementary Table S3. Risk of bias for each included study, reporting item by item assessment. Studies are listed in alphabetical order and stratified by study design.

	**Cross-sectional studies (using the New-Castle Ottawa Scale adapted NOS-xs)**							
**Author, year [ref]**	**Item 1**	**Item 2**	**Item 3**	**Item 4**	**Item 5**	**Item 6**	**Total stars**	**Overall quality**
Abeliotis K., et al., 2016 [47]	0	0	1	1	0	0	**2**	High risk of bias
Aloysius N., et al., 2025 [48]	1	1	1	1	0	0	**4**	Moderate risk of bias
Arrazat L., et al., 2024 [49]	1	0	2	2	2	1	**8**	Low risk of bias
Bender K.E., et al., 2022 [50]	1	0	1	1	2	1	**6**	Moderate risk of bias
Carroll N., et al., 2021 [51]	0	0	1	2	1	1	**5**	Moderate risk of bias
Casucci M., et al., 2026 [44]	1	0	2	2	0	0	**5**	Moderate risk of bias
Chen H., 2019 [52]	1	0	1	1	1	1	**5**	Moderate risk of bias
Di Costanzo G. et al., 2025 [53]	1	0	1	2	2	1	**7**	Low risk of bias
Gonçalves C., et al., 2025 [54]	0	0	2	2	1	1	**6**	Moderate risk of bias
Karunasena GG., et al., 2021 [55]	1	0	1	1	1	1	**5**	Moderate risk of bias
Meixner O., et al., 2020 [56]	1	0	1	1	0	0	**3**	High risk of bias
Mengi Çelik O., et al., 2025 [57]	0	0	2	2	1	1	**6**	Moderate risk of bias
Morosan E., et al., 2024 [58]	0	0	1	1	0	0	**2**	High risk of bias
Rodgers RF., et al., 2021[59]	0	0	1	1	1	1	**4**	Moderate risk of bias
Romani S., et al., 2018 [60]	1	0	1	1	0	0	**3**	High risk of bias

Yetkin Özbük RM., et al., 2022 [61]	0	0	1	1	0	0		**2**			High risk of bias
	**Pre-post interventions (using the Joanna Briggs Institute for quasi experimental studies)**										
	**Item 1**	**Item 2**	**Item 3**	**Item 4**	**Item 5**	**Item 6**	**Item 7**	**Item 8**	**Item 9**		**Overall quality**
Adam M., et al., 2015 [43]	Yes	NA	NA	No	Yes	No	Yes	No	Yes		Moderate risk of bias
Burrington C.M., et al., 2020 [64]	Yes	NA	NA	No	Yes	Yes	Yes	No	No		Moderate risk of bias
Cemali O., et al., 2025 [65]	Yes	NA	NA	No	Yes	No	Yes	No	Yes		Moderate risk of bias
Garcia A.L. et al., 2017 [66]	Yes	NA	NA	No	Yes	No	Yes	Yes	Yes		Low risk of bias
Garcia T et al., 2021 [67]	Yes	NA	NA	No	Yes	unclear	NA	No	Yes		Moderate risk of bias
Laila A., et al., 2024 [68]	Yes	NA	NA	No	Yes	Yes	NA	Yes	Yes		Low risk of bias
Marconi S. et al., 2026 [45]	Yes	NA	NA	No	Yes	Yes	NA	Yes	Yes		Low risk of bias
Polak R., et al., 2018 [69]	Yes	NA	NA	No	Yes	Yes	NA	Yes	Yes		Low risk of bias
Remolina I., 2025 [70]	Yes	unclear	unclear	No	Yes	unclear	Yes	Yes	Yes		Moderate risk of bias
	**Qualitative studies (using the JBI for Qualitative Research)**										
	**Item 1**	**Item 2**	**Item 3**	**Item 4**	**Item 5**	**Item 6**	**Item 7**	**Item 8**	**Item 9**	**Item 10**	**Overall quality**
Finkelstein A., et al., 2025 [46]	Unclear	Yes	Yes	Yes	Yes	Unclear	Unclear	Yes	Yes	Yes	Moderate risk of bias
Nonomura M., et al. 2019 [63]	Unclear	Yes	Yes	Yes	Yes	Unclear	Unclear	Yes	Yes	Yes	Moderate risk of bias
	**Cluster randomized pilot trial (using Revised Cochrane Risk-of-Bias tool for randomized trial – 2)**										
	**Item 1**	**Item 2**	**Item 3**	**Item 4**	**Item 5**	**Item 6**					**Overall quality**

Metcalfe J. J., et al, 2022 [62]	Some concerns	Some concerns	Low	Some concerns	Some concerns	Low	Some concerns
